# Insights on Oligometastatic Non-Small-Cell Lung Cancer

**DOI:** 10.3390/cancers17152451

**Published:** 2025-07-24

**Authors:** Augusto Valdivia, Pau Mascaro-Baselga, Clara Salva-de Torres, Abraham Geng-Cahuayme, Sara Torresan, Jesus Yaringaño, Ilaria Priano, Patricia Iranzo, Nuria Pardo, Laura Masfarre, Oriol Mirallas, Karen Farfan, Susana Cedres, Pedro Rocha, Alex Martinez-Marti, Enriqueta Felip

**Affiliations:** 1 Medical Oncology Department, Vall d’Hebron Hospital Universitari/Vall d’Hebron Institute of Oncology (VHIO), 08035 Barcelona, Spain; 2 Radiotherapy Oncology Department, Vall d’Hebron Hospital Universitari/Vall d’Hebron Institute of Oncology (VHIO), 08035 Barcelona, Spain; 3 Department of Medical Oncology, Centro di Riferimento Oncologico di Aviano (CRO), 33100 Udine, Italy; 4 Medical Oncology Department, Hospital Nacional Edgardo Rebagliati Martins, Lima 15072, Peru

**Keywords:** oligometastasis, non-small-cell lung cancer, local ablative therapies, immunotherapy, targeted therapy, biomarkers

## Abstract

Oligometastatic non-small-cell lung cancer (OMD-NSCLC) has emerged as a biologically and clinically distinct subtype of advanced disease, characterized by limited metastatic burden and a more indolent course. In this narrative review, we examine the current definition of OMD-NSCLC, diagnostic tests, possible biomarkers, and current therapeutic strategies. Biological insights highlight the role of microRNAs in differentiating true oligometastatic state from polymetastatic disease. The main local ablative therapies (LAT) include surgery and radiotherapy. The integration of LAT with systemic therapies has been explored in clinical trials, yielding promising but occasionally inconsistent results. As the therapeutic landscape of OMD-NSCLC patients continues to evolve, refining definitions, identifying predictive biomarkers, and individualizing care are essential steps toward achieving the potential of radical-intent therapy.

## Simple Summary

Non-small-cell lung cancer (NSCLC) is often diagnosed at advanced stages, where treatment options are usually limited. A subgroup of patients presents with only a few metastases, a condition known as oligometastatic NSCLC (OMD-NSCLC). These patients may benefit from more aggressive treatment aimed at long-term control. This review explains how OMD-NSCLC is defined, how it can be identified through imaging and clinical features, and what treatments are available. Local treatments such as surgery or radiotherapy can be used to treat all tumors. New research also explores the biology of the disease, including genetic and molecular markers that may predict better outcomes. The combination of local therapies with immunotherapy or targeted therapy is being explored in clinical trials. Understanding who may benefit most from these strategies is key to improving survival and quality of life in OMD-NSCLC.

## 1. Introduction

Non-small-cell lung cancer (NSCLC) represents a major global health issue, being a leading cause of cancer-related morbidity and mortality. Most NSCLC cases are diagnosed at later stages of disease, where radical intent therapy is difficult to attain [1,2,3]. The first time that metastatic cancer was proposed to be curable was in 1968 [4]. In 1995, Hellman and Weichselbaum hypothesized the existence of an intermediate state between localized and widespread metastatic disease, characterized by a limited number of metastases. They defined the concept of oligometastasis (from the Greek “*olígos*”, meaning few) and hypothesized that, at this stage, the disease could potentially be curable, provided that local ablative therapies (LAT) to all sites of disease were feasible and safe [5].

Oligometastatic NSCLC (OMD-NSCLC) is now considered a distinct clinical entity within NSCLC, associated with a more indolent disease course and better prognosis, opening the possibility for a multimodal, radical, and potentially curative treatment [6,7].

In this review, we examine the current state of OMD-NSCLC, exploring its definition, biological insights, diagnosis, and treatment strategies, including both local and systemic approaches.

## 2. Methods: Literature Search Strategies

To elaborate on this review, we conducted a comprehensive literature search using the PubMed/MEDLINE database, complemented by manual reference screening and selection of relevant articles. The search covered studies published between January 1995 and April 2025, especially prioritizing studies published after 2015. However, select key foundational papers published prior to this date were also included when considered essential due to their historical and/or conceptual importance.

The search strategy used Boolean operators (e.g., AND/OR/NOT) to combine of the following MeSH terms and keywords: “oligometastasis” or “oligometastatic”; “non-small-cell lung cancer” or “NSCLC”; “definition”; “biology”; “molecular”; “miRNA”; “immune microenvironment”; “local ablative therapies”; “radiotherapy”; “stereotactic ablative radiotherapy” or “SABR”; “stereotactic beam radiotherapy” or SBRT”; “stereotactic radiosurgery” or “SRS”; “radiofrequency ablation” or “RFA”; “surgery”; and “metastasectomy”. Filters were applied to restrict articles to human studies, English-language publications, and peer-reviewed journal publications. Priority was given to randomized clinical trials, systematic reviews or meta-analyses, well-designed prospective studies, high-quality review articles in top-tier journals, clinical guidelines or consensus statements (e.g., ESMO, NCCN, and ESTRO-ASTRO), or articles deemed relevant by the authors for their contributions to the biological understanding of oligometastatic disease.

## 3. Diagnosis and Classification of OMD-NSCLC

*Synchronous OMD* concerned by the lack of a uniform definition and staging procedures used to diagnose synchronous (or de novo) OMD-NSCLC, i.e., oligometastatic state at disease presentation, the European Organization of Research and Treatment of Cancer (EORTC) conducted a consensus report composed of 35 thoracic oncology experts. The definition was considered crucial when radical treatment could be safely implemented across all anatomic sites, allowing the possibility of long-term disease control. The consensus confined synchronous oligometastatic disease to a maximum of five metastases in a maximum of three organs, excluding diffuse serosal metastases (meningeal, pericardiac, pleural, mesenteric) and bone marrow involvement. Fludeoxyglucose F 18–positron emission tomography–computed tomography (^18^F-FDG PET-CT) and brain imaging, preferably magnetic resonance imaging (MRI), were mandated to be carried out for staging purposes. Pathological confirmation should be performed on metastatic sites if feasible, and mediastinal staging was also recommended [8].

The European Society for Radiotherapy and Oncology (ESTRO) and the American Society for Radiation Oncology (ASTRO) also carried out a consensus statement broadening the concept of synchronous OMD disease to other solid tumors. Following a systematic literature review and a Delphi survey, the panelists concluded that the maximum number of metastases required to define OMD should be limited by the capacity to safely deliver curative intent radiation to said lesions. However, since most of the literature studied reported a threshold of up to five metastatic sites, it was decided to restrict the number to five [9]. The main limitations of both consensuses come from the absence of biological evidence supporting a maximum number of metastases or a maximum lesion size that can be treated with LAT. This was especially important for patients with polymetastatic disease, as novel local techniques can treat multiple lesions with radical intent. Moreover, prospective information on survival outcomes and safety was limited. The E^2^-RADIatE OligoCare cohort (NCT03818503), a joint effort of the ESTRO and EORTC, aims to answer these inquiries [9].

The novel ninth edition of the TNM staging classification of lung cancer has divided the M1c category into M1c1 (multiple metastases in one organ system) and M1c2 (multiple metastases in multiple organ systems). M1a and M1b are still unchanged. This continues to reflect the prognostic implications of metastatic sites in median overall survival: M1a 1.3 years, M1b 1.2 years, M1c1 1 year, and M1c2 0.6 years. TNM’s M subcommittee found a statistically significant difference in OS when a cut point of seven lesions was carried out. However, nondichotomized analysis indicated a progressive worsening of survival with a greater number of metastatic lesions. Furthermore, the data available did not include LAT exposure to patients [10].

*Metachronous OMD* entity is characterized by the discovery of a limited number of metastatic lesions during or after primary tumor treatment, with a six-month timeframe being the most accepted. Contrary to de novo OMD-NSCLC, there are multiple subcategories of the metachronic state, depending on whether the patient is receiving active oncologic treatment (oligorecurrence vs. oligoprogression) or there was a prior diagnosis of oligometastatic or polymetastatic disease (repeat vs. induced OMD), among others [11,12].

The European Society of Medical Oncology (ESMO) guidelines for advanced NSCLC integrate the previously mentioned definitions of OMD-NSCLC and their different characteristics. They also recommend carrying out ^18^F-FDG PET-CT and brain imaging to stage synchronous and metachronous OMD-NSCLC [13].

A summary of different definitions of OMD-NSCLS is depicted in Table 1.

## 4. Clinical Factors Impacting the Prognosis of OMD-NSCLC

Multiple studies have identified risk factors of worse prognosis that may help identify patients with oligometastatic disease who would not benefit from a radical treatment approach with LAT.

A retrospective systematic review, encompassing 49 studies and 2176 patients with NSCLC and 1–5 metastases treated with LAT to all metastatic sites, found that definitive treatment of primary tumor, negative nodal status, and disease-free interval (DFS) of at least 6–12 months were all associated with improved OS [21]. Similarly, a meta-analysis pooling data from 757 patients with OMD-NSCLC (defined as up to 5 metastases and all treated with either radiotherapy or surgical excision) reported a median OS of 26 months, with a 1-year survival rate of 70.2% and a 5-year OS of 29.4%. Factors associated with longer survival included metachronous metastases (as opposed to synchronous), N0 disease stage, and adenocarcinoma histology [22].

A retrospective study of 180 patients with oligometastatic NSCLC limited to a single organ (up to four metastases) and treated with LAT yielded comparable results. Factors associated with better outcomes included adenocarcinoma histology, T1a stage, Eastern Cooperative Oncology Group Performance Status (ECOG) of 0–1 (versus 2), and N0-2 stage (versus N3) [23]. In a comparable manner, a prospective study of 186 patients with synchronous oligometastatic NSCLC (≤5 metastases at diagnosis) found that ECOG ≥2, N2-3 stage, squamous histology, and metastasis to more than one organ were all associated with poorer OS. Notably, the number of metastatic lesions was not prognostic in this study [24].

Taken together, these findings suggest that factors other than the number of metastases are important for predicting outcomes in low-volume, metastatic NSCLC. Particularly, negative nodal status and a low rate of progression (with >6 months of DFS or a metachronous pattern of metastatic spread serving as surrogate markers) are strong favorable prognostic factors [25].

## 5. Biological Insights and Potential Biomarkers in OMD NSCLC

The existing OMD-NSCLC categories have limitations. Mainly, they ignore important biological aspects such as tumor aggressiveness, temporal dynamism, or translational features. This is significant, as a large proportion of patients treated with LAT will nevertheless have an unfavorable clinical course, most likely due to the presence of undetected micrometastatic disease. In that sense, solely relying on the number of metastases appears insufficient to define a genuine oligometastatic state [26,27].

Time appears to be essential in the oligometastatic equation, a finding consistent across various tumor types. In a prospective study of 63 patients with different oligometastatic cancers undergoing lung metastasectomy with curative intent, a slow pace of metastatic progression (defined as fewer than 0.6 new metastases per year after surgery) was associated with significantly longer OS compared to those with a high rate of progression (more than 3.6 new metastases per year) [28]. Likewise, in a prospective study of 1001 patients with metastatic colorectal cancer undergoing liver resection, a short DFS (<12 months from primary tumor resection to liver metastases development) was strongly correlated with poorer long-term outcomes [29]. Szturz and Vermorken have proposed the term argometastasis (from the Greek “*argos*”, meaning slow) instead of oligometastasis to better define the oligometastatic state, defining the associated clinical characteristics [30].

Variations in the micro-RNA (miRNA) expression profiles may also explain differences in the biology of metastases. miRNAs are small, non-coding RNA molecules that regulate gene expression at the post-transcriptional level, namely through messenger RNA (mRNA) interference and silencing [31].

In patients with resected lung metastases from multiple solid tumors, Lussier et al. identified differential expression profiles in 40 miRNAs between patients with different rates of progression. Patients with rapidly progressing tumors displayed downregulation of most of the studied miRNA, many of which have been implicated in tumor suppression functions. Furthermore, the authors demonstrated that the downregulation of two of these miRNAs (miR-328 and miR-502-5p) was also associated with a fast rate of tumor progression in an independent validation cohort [28].

Lussier et al. also analyzed the miRNA expression patterns in 29 patients with solid tumors (6 patients with NSCLC) and five or less metastatic lesions. Patients were classified into two groups according to their response after radiotherapy LAT: polymetastatic and oligometastatic patients. Polymetastatic patients were defined as those that suffered from the appearance of five new metastatic lesions in less than four months after treatment administration or progression in a body cavity that implied diffuse metastatic disease (i.e., pericardial, pleural, cerebrospinal, or ascitic fluid). Tumors from patients with polymetastatic progression harbored an enhancement in the expression of miRNA-200c, implicating that this miRNA may cause a transition to a polymetastatic phenotype [32,33]. Additionally, another study investigated the mRNA and miRNA expression in resected liver metastases from 134 colorectal cancer patients. Based on the expression profile, three molecular subtypes of colorectal liver metastases with prognostic value were identified: immune, canonical, and stromal, with 10-year OS rates of 64%, 37%, and 20% [34].

In the TRACERx Renal study, which included 100 patients with clear-cell renal cell carcinoma, matched biopsies from primary tumors and metastatic lesions were analyzed. Losses of 9p and 14q were identified as important drivers for metastasis development [35]. Regarding the 14q region, another study has shown that the expression of several miRNAs encoded on the 14q32 locus is associated with a better prognosis in patients with metastatic cancer [36]. Moreover, Oshima et al. demonstrated that the expression of miRNAs from this locus suppresses metastatic spread in preclinical models [37].

Concerning genomic alterations, Sutera et al. explored the mutational landscape of 294 patients with metastatic castration-sensitive prostate cancer and compared three subgroups with different disease burdens (biochemical recurrence only, low volume, or large volume of metastatic disease). The investigators observed significant differences in driver mutations in *TP53*, *WNT*, cell cycle genes, and the PI3KCA/AKT/mTOR pathway between groups, with higher mutation frequency as metastatic burden increased. Patients with low-volume metastatic disease and *TP53* mutation exhibited clinical outcomes like those with high-volume metastatic disease, suggesting that genomic characteristics of the tumor may be more relevant for prognosis than disease burden [38]. Currently, the relationship between genomic alterations and clinical outcomes in OMD-NSCLC has not been clearly established [39].

In conclusion, the molecular features of tumors may provide a more accurate prediction of metastatic phenotype and outcomes than the number of metastatic sites. The evidence discussed raises questions about the adequacy of the current definition of oligometastatic disease. While an ideal definition might eventually be based on molecular criteria, our current understanding is insufficient to robustly establish such a definition.

## 6. Local Ablative Therapies

The optimal local management of OMD-NSCLC is currently unknown, as clinical trials comparing different LAT are lacking. The choice of LAT can vary widely and should be discussed in a multidisciplinary team (MDT), considering clinical characteristics (ECOG, comorbidities, expectations), metastases features (size, number of lesions, and resectability), and treatment availability, as well as professional training in medical centers. The main LAT administered to patients with OMD-NSCLC are surgery and radiotherapy [13].

Concerning these two LAT, Griffoen et al. carried out a retrospective analysis of 67 patients with synchronous OMD-NSCLC that received radical therapy with either surgical resection or RT (biological dose equivalent to 13 fractions of 3 Gray). The median OS of the cohort was 13.5 months. However, treatment of the primary tumor, either with a smaller RT planning target volume (lower than 639 cc) or surgical resection, was associated with improvement in survival [40]. Improvement in survival with LAT was also described in an observational study including 91 patients diagnosed with OMD-NSCLC between 2008 and 2016. Radical treatments for primary tumor and metastases included surgical excision, radiotherapy, or radiofrequency ablation (RFA) [41].

*Surgical resection:* As previously mentioned, surgical indication on metastases depends on localization, size, number of lesions, secondary symptoms, risks of surgery, and the patients’ basal status and preference.

*Primary tumor and lung metastases:* The International Registry of Lung Metastases reported outcomes of 5206 patients with pulmonary metastases from epithelial tumors, sarcomas, germ cell tumors, and melanomas. The most common type of resection was wedge resection (61.91%), followed by lobectomy (19.77%), segmentectomy (8%), and pneumonectomy (2.37%). Other types of resections were carried out in 7.95% of patients. Complete resection was achieved in 86% of patients, and perioperative mortality occurred in only 1% of cases. Chemotherapy was administered to 1986 patients; 57% received it before surgical intervention. The 5-year OS rate in patients with complete resection was 36%, which increased to 45% in patients with disease-free intervals (DFI) greater than 36 months [42]. The efficacy of lung metastasectomy was also shown in a more recent cohort of patients. In this study, factors associated with improvement in prognosis also included complete resection, germ cell tumors, and DFI greater than 36 months. Importantly, surgical approach and presence of lymph node metastases did not appear to influence survival [43]. Furthermore, a retrospective analysis of patients with NSCLC and simultaneous secondary malignant lesions (either T4 or M1a) showed a potential benefit of surgery of the primary tumor and resection of the solitary pulmonary lesion, with median survival of 79 months for patients with T4 tumors and 84 months for patients with contralateral lung metastasis [44]. There is also growing evidence supporting the resection of the primary tumor as part of the management of OMD-NSCLC. In a propensity-matched analysis of 12,215 patients with single-organ OMD-NSCLC, improvement in median OS was seen in patients who underwent surgery of the primary tumor (349 patients): 36.8 months vs. 20.8 months (HR 0.67, CI 95% 0.56–0.80) [45].

*Brain metastases:* two trials have demonstrated the benefit of surgical resection of brain metastases. Patchell et al. conducted a prospective randomized trial to compare surgical resection of single brain metastasis (BM) followed by whole brain RT (WBRT) vs. WBRT alone in patients with brain metastases from solid tumors. A total of 48 patients were analyzed; 77% had NSCLC. The rate of brain recurrence was significantly lower in the surgical group (25% vs. 52%). Median OS was superior in the surgical group, with an RR of 2.2 (95% CI 1.2–4.1, *p* < 0.001). Patients in the surgical resection group also benefited from an improvement in quality of life [46]. Similarly, Vecht et al. also demonstrated improvement in overall survival for patients with metastatic solid tumors (50% had NSCLC) and solitary brain metastases that underwent surgical resection followed by WBRT. Factors that predicted better outcomes included younger age and stable extracranial tumor activity during the previous three months [47].

Retrospective data on surgically removed BM have shown that factors negatively affecting survival include incomplete resection, infratentorial lesions, no resection of the primary tumor, and lymph node involvement of the primary disease. Interestingly, the diameter of the resected metastatic lesion seems to not influence survival [48,49,50,51].

The European Association of Neuro-Oncology–European Society of Medical Oncology (EANO-ESMO) guidelines for the management of BM from solid tumors recommend surgical resection of BM in patients presenting with raised intracranial pressure or neurological impairment. It is assumed that patients with more than one BM could also benefit from surgery if a total resection is feasible and safe [52].

*Adrenal metastases:* contrary to BM, most of the evidence regarding surgical management of adrenal metastases (AM) comes from retrospective analysis of small cohorts of patients. Historical approaches for AM resection included lumbotomy, laparotomy, and phrenotomy during thoracotomy. Currently, laparoscopic adrenalectomy is the surgical method of choice [53,54,55,56].

Krumeich et al. conducted a multi-institutional cohort study of 122 patients with lung cancer who underwent surgical resection of AM. Of these, 38.5% of patients had synchronous oligometastatic, and 23% had extra-adrenal metastases. In this cohort of patients, median OS was 47 months, with a 5-year OS rate of 35.2%. In a multivariate analysis, factors associated with worse OS included the presence of extra-adrenal metastases at the time of adrenalectomy (HR 3.52, *p* = 0.007), small-cell histology (HR 15, *p* = 0.04), and prior lung radiation (HR 3.37, *p* = 0.02) [57].

Surgery in other locations, such as bone and liver, should be carried out by surgeons with extensive experience, as recommended by the ASTRO-ESTRO clinical practice guideline [58].

*Radiotherapy:* Different RT modalities are now available and have been used in oligometastatic disease in a wide range of lesions. Stereotactic beam radiotherapy (SBRT) or stereotactic ablative radiotherapy (SABR) is a precise form of external RT that delivers high doses of ionizing radiation via image guidance on an extracranial lesion. When applied to intracranial lesions, it is called SRS, with approximately 1 mm accuracy, and is typically used on patients with a limited number of lesions. RT offers interesting advantages compared to surgery, as it is a non-invasive and well-tolerated technique. Its use is strongly advised when multiple organs are involved or systemic therapy breaks must be minimized [58].

SABR-COMET was an open-label phase 2 randomized international study that included patients with 1–5 metastatic lesions and a controlled primary tumor, including breast, colorectal, lung, prostate, and others. Patients in the control arm received standard palliative RT to alleviate symptoms, while patients in the SABR arm received SABR to all metastatic sites. Both arms underwent standard-of-care (SOC) systemic therapy as indicated. The primary endpoint was OS. A total of 99 patients were enrolled, 18 patients with lung cancer. Approximately 70% of patients had one or two metastases. Lung (47%) and bone (34%) were the most frequent localizations, followed by liver (10%) and adrenal (5%) metastases. After a median follow-up of 28 months, the SABR arm demonstrated a 13-month improvement in median OS [59]. In the long-term analysis, the effects of SABR on OS were larger, with a median OS benefit of 22 months (28 months in the control arm vs. 50 months in the SABR arm, HR 0.47 (CI 95% 0.27–0.81)). No differences were found in quality-of-life scores between both arms. However, overall rates of grade 2 or more adverse events were 9% in the control arm and 29% in the SABR arm, with three deaths probably related to treatment in the latter group [60].

In interim analyses of the OligoCare study, cancer-specific dose and fractionation schedules for SBRT were heterogeneous. The most frequently treated sites are non-vertebral bone (22.8%), followed by lung (21%) and distant node metastases (19%). Importantly, acute toxicity at 6 months was low (0.5% of grade ≥ 3, two fatal cases) [61,62].

The role of RT in the management of oligoprogressive disease has been explored in two randomized clinical trials. The CURB trial included patients with breast cancer or NSCLC after first-line therapy with up to five increasing lesions that were randomized to SOC plus SABR targeting the progressive sites versus SOC alone. A total of 106 patients were enrolled (59 patients with NSCLC). The median PFS for NSCLC patients was 10.0 months in the SABR group, while the PFS reported for the control arm was 2.2 months. Notably, no differences were observed for breast cancer patients [63]. Recently, results from the phase 2 STOP trial, with a similar design, were published. They included 90 patients with 1–5 oligoprogressive metastases on first-line treatment, 44% with primary lung lesions, and randomized them to receive SOC with or without SABR to all progressing lesions. No differences in PFS nor OS were observed, although lesional control was superior in the SABR arm (70% vs 38%) [64].

*Primary tumor and lung metastases:* A retrospective study of 34 patients (11 lung cancer cases) with oligo-recurrent or synchronous lung metastases treated with SABR (BED10 ≥ 75 Gy) from 2004 to 2014 in Japan. Most patients presented with only one metastatic lesion (*n* = 29). The 2-year OS rate of the whole patient cohort was 65.7%, without late grade ≥ 3 adverse events reported. Notably, the 2-year OS rate was 68.5% in the oligo-recurrence group and 50% in the synchronous disease group [65].

In patients with NSCLC, De Rose et al. conducted an observational study including patients diagnosed with OMD-NSCLC with lung metastases measuring 5 cm or less treated with SABR. Sixty patients with ninety lung lesions were analyzed. Most patients had one or two lesions (67% and 22%, respectively). Only three patients had four lesions, and four patients had three lesions. The most common SABR dose prescription was 48 Gy in four fractions (78%), followed by 60 Gy in eight fractions (16%). At 2 years, the local control rate was 88.9%. Overall survival rates were 94.5% and 74.6% at 1 and 2 years. Eighty percent of patients had grade 1–2 lung toxicity. One patient had a grade 3 lung toxicity in the first 6 months, and one patient presented with a late pulmonary toxicity after 1 year. More serious adverse events, such as hemorrhage or broncho-esophageal fistula, have been related to central lesions, defined as being within 2 cm from midline structures [66,67].

*Brain metastases:* As mentioned earlier, BM can be treated with RT alone if surgery is not feasible. To reduce recurrence rates after neurosurgical resection, SRS to the resection cavity was evaluated in two randomized trials. Brown et al. compared post-operative WBRT or SRS in 194 patients, and they reported no difference in OS, but a more frequent decline of cognitive function after WBRT [68]. Mahajan et al. compared post-operative SRS or observation, describing a lower local recurrence in the SRS arm [69].

Surgery and SRS have not been directly compared in prospective randomized trials. However, retrospective data suggest that SRS is a well-tolerated alternative with similar outcomes. A retrospective review of patients with one to four brain-only OM-NSCLC at six hospitals in Japan between 1996 and 2008 included patients treated either with SRS or stereotactic RT (SRT, a fractionated form of SRS) delivered in four to five fractions. Eleven patients presented with synchronous disease, while fifty patients had an oligo-recurrence. Forty-five patients were treated with SRS and sixteen patients with SRT. Thoracic lesions were staged as I-II (44%) and III (56%) and treated either with surgery (70%) or RT with or without concurrent chemotherapy (30%). The median OS was 26 months. The 2-year and 5-year OS rates were 60.7 and 15.2%, respectively. The median OS was longer for patients with an oligo-recurrence compared to synchronous disease (41 months vs. 18 months) [70].

ASTRO’s clinical practice guideline recommends SRS for patients with an ECOG-PS of 0–2 with up to 4 BM and for patients with an ECOG-PS of 0–2 and 5–10 BM as a conditional recommendation. The diameter of the lesion is also considered, recommending a single-fraction SRS for lesions measuring < 2 cm and multifraction SRS in lesions measuring 3 to 4 cm. For lesions measuring more than 4 cm, multifraction SRS would be an alternative if surgery is not feasible, although, for a tumor size of more than 6 cm, SRS is discouraged [71].

*Adrenal metastases:* although AM has a well-established surgical approach, SABR has gained relevance in the past years. A meta-analysis of 39 studies included 1006 patients, of whom 65.7% had a primary lung tumor and 73% had metachronous lesions. Reported local control rates were 82% at 1-year and 63% at 2-year follow-up, with a grade 3 toxicity rate of 1.8% [72]. Promising local control was observed in a retrospective, single-center analysis that included 56 patients with oligometastatic (1–5 lesions) or oligoprogressive NSCLC who were treated for AM with SABR. Twenty-eight patients were excluded due to the use of palliative doses. Out of the 28 patients analyzed, most patients were diagnosed with NSCLC (46%) and presented with a single AM (61%). The overall response rate based on RECIST criteria was 86% (CR  =  29%, PR  =  57%), with a local control rate at 2 years of 85%. The most common acute toxicities were fatigue and gastrointestinal symptoms. All of them, grade 1 or 2 [73]. Another retrospective review of 31 patients who underwent SABR for AM included 45.2% of patients with lung cancer and 84.8% of metachronous lesions. Results showed an overall response rate of 64.6% (CR = 32.3%, PR = 32.3%) and 1- and 2-year local control rates of 96.5% and 92.6%, respectively. The most common acute toxicities were nausea, abdominal pain, vomiting, and asthenia, all of which were grade 1–2. No grade 3 or more were reported [74].

*Liver metastases:* The role of RT in the local management of liver metastases is expanding. An international registry included 427 patients, of which 12.2% were diagnosed with lung cancer (*n* = 52). Median OS of patients with hepatic metastases volume < 40 cm^3^ was longer than those with volumes ≥ 40 cm^3^ (25 vs. 15 months). Similarly, a BED10  ≥ 100 Gy was associated with improved OS, with a median OS of 27 months compared to 15 months for BED10  < 100 Gy. Local control rates were better for a BED10 ≥100 Gy (87.5% vs. 71.8% at 1 year) and a tumor volume of less than 40 cm^3^ (52 vs. 39 months) [75]. Similar results of local control rates were reported from a phase 1/2 trial including 47 patients (10 with lung cancer) with 1–3 hepatic lesions and a maximum of 6 cm of diameter who received an escalating dose of 36 to 60 Gy. Local control rates after 1 and 2 years were 95% and 92%, respectively [76].

Until now, evidence has offered promising data and some conflicting results on the use of SABR in OMD-NSCLC. Multiple phase 3 trials are currently ongoing, trying to give answers to several questions and controversies, including the acceptable number of lesions for radical treatment, the timing of local therapies, and the optimal combination with systemic therapy [77]. SABR-COMET10 is a phase 3 trial designed to include patients with a maximum of 10 oligometastatic lesions (NCT03721341), while SABR-COMET3 includes patients with one to three lesions (NCT03862911). SARON (NCT02417662) is a confirmatory phase 3 trial assessing the efficacy and safety of standard chemotherapy combined with SABR or conventional RT in patients with OMD-NSCLC.

*Other local ablative therapies*: Radiofrequency ablation (RFA) was first applied to hepatocellular carcinoma. This technique consists of the placement of a radiofrequency electrode that causes heating of the surrounding tissue and focal coagulation necrosis by delivery of energy in the form of an alternating electrical current [78]. The RAPTURE prospective study included 106 patients with lung tumors of 3.5 cm or smaller unfit for surgery and RT or chemotherapy that underwent RFA and were followed for 2 years. A total of 75 patients presented a complete response of target tumors lasting at least 1 year. Moreover, OS was 70% at 1 year and 48% at 2 years in patients with NSCLC. They reported 27 cases of pneumothorax and 4 pleural effusions that needed pleural drainage [79]. In the oligorecurrent setting, Kodama et al. conducted a study on 44 patients with oligorrecurrent NSCLC that underwent RFA, with a total of 55 RFA sessions. The OS rates were 98%, 73%, and 56% at 1, 2, and 3 years. They reported three cases of pneumothorax requiring pleurosclerosis or surgical suture [80].

Microwave ablation (MWA) has also been applied in patients with OMD-NSCLC, showing promising control rates in retrospective case series [81]. In patients receiving first-line EGFR inhibitors, the addition of MWA showed a median duration of response of 29 months. Randomized clinical trials are lacking [82].

Further prospective studies are needed to define the role of RFA as an alternative option to surgery and RT. Other unconventional techniques with limited experience and data include microwave ablation and cryoablation, already used on stage I NSCLC, with comparable results to other ablation techniques [83]. Laser ablation has not been used in this field, while irreversible electroporation failed to show local control in lung cancer in the ALICE trial [84].

## 7. Systemic Therapy for the Management of OMD-NSCLC

Historically, three clinical trials have propelled the development of LAT in addition to systemic treatment as part of the therapeutic paradigm in patients with OMD-NSCLC.

De Ruysscher et al. conducted a single-arm prospective phase II clinical trial including patients with advanced NSCLC with fewer than five metastases at diagnosis amenable to surgery or RT. The primary endpoint was OS at 2 and 3 years. Thirty-nine patients were eligible for analysis. The most frequent sites of metastases were the brain (43.9%), bone (17.9%), and adrenal gland (10.3%). The primary tumor was treated with concurrent (53.8%) or sequential (38.5%) chemoradiotherapy. Concurrent chemotherapy regimens included cisplatin–vinorelbine (66.6%) and cisplatin–etoposide (33.3%), while sequential treatment regimens primarily used cisplatin–gemcitabine (73.3%). Adrenal metastases were surgically removed (except in one case), bone metastases were treated with radiotherapy, and most brain metastases received stereotactic radiosurgery (SRS). After a median follow-up of 28.3 months, the median PFS was 12.1 months, and the median OS was 13.5 months. The 1-year and 3-year OS rates were 56.4% and 17.5%, respectively. Acute grade 3 esophagitis occurred in 15% of patients, and no grade ≥ 3 toxicities were reported following LAT to distant metastases. Notably, no patients received immunotherapy upon progression [27,85].

Gomez et al. conducted a multicenter, randomized, controlled, phase II trial including stage IV NSCLC patients with ≤ 3 metastases. After receiving four cycles of platinum-based doublet therapy or ≥3 months of EGFR/ALK-targeted therapy as induction, patients without disease progression were randomized to LAT (with or without maintenance therapy) versus maintenance alone. Of the 49 patients randomized, 80% had adenocarcinoma, 84% were EGFR or ALK wild-type, and 94% had synchronous metastases. The most common metastatic sites included the brain (*n* = 13), bone (*n* = 10), adrenal gland (*n* = 8), pleura (*n* = 7), and lung (*n* = 6). LAT included hypofractionated radiotherapy/SABR (48%), surgery plus radiation (24%), chemoradiotherapy (8%), combined hypofractionated RT and chemoradiotherapy (12%), and surgery to all sites (4%). The trial was closed prematurely due to a significant difference in median PFS in the LAT arm (11.9 vs. 3.9 months, HR 0.35). Adverse events were comparable across arms; there were no grade 4 adverse events or treatment-related deaths. Grade ≤ 3 events were reported in five patients in the LAT arm, including two cases of grade 3 esophagitis, one case of grade 2 esophagitis, one case of anemia induced after RT to the spleen, and one pneumothorax secondary to a rib fracture after a lung SABR [86]. A significant OS benefit was also observed (41.2 vs. 17.0 months, *p* = 0.017) [87].

Finally, Iyengar et al. conducted a two-arm randomized phase II trial comparing maintenance systemic therapy with or without SABR in patients with metastatic NSCLC who had received 4–6 cycles of first-line platinum-based chemotherapy, achieving stable disease or partial response. Patients with *EGFR*- and *ALK*-mutated NSCLC were excluded. This study was also stopped following positive findings at interim analysis. Of 29 randomized patients, the median PFS was 9.7 months in the SABR arm versus 3.5 months in the control arm (HR 0.30, 95% CI 0.11–0.82, *p* =0.01). Median OS was not reached in the SABR group, while median OS in the control arm was 17 months, including crossover of two patients. Treatment-related toxicity was comparable. There were two grade 3 toxicities and one grade 4 probably related to treatment in the control arm, while the SABR and maintenance arm reported four cases of grade 3 toxic effects attributed to treatment [88].

Importantly, the introduction of targeted therapy and immunotherapy has transformed the treatment paradigm in patients with advanced NSCLC and is currently the standard of care. Examining their role in the therapeutic framework of OMD-NSCLC is essential [89].

*OMD-NSCLC without targetable molecular alterations (TMA):* with the advent of immunotherapy, preclinical evidence highlighted the synergy between radiotherapy and immune-checkpoint inhibition [90]. Several retrospective studies have suggested that adding immunotherapy-based systemic therapy to LAT improves survival outcomes in patients with OMD-NSCLC [91,92,93,94]. Furthermore, two single-arm phase 2 clinical trials have shown that administering SABR to a single lesion may enhance subsequent response to PD-1 inhibition, particularly in patients with PD-L1-positive tumors [95,96].

The ongoing LONESTAR trial (NCT03391869) is a randomized phase III study in immunotherapy-naïve patients (prior chemotherapy allowed). Patients receive two cycles of nivolumab and ipilimumab as induction therapy, and those without progression are randomized to continue immunotherapy with or without LAT (cohort 1). At the ASCO annual meeting 2022, results from patients with progressive disease after induction treatment were presented. Of 194 patients, 72 (37%) experienced disease progression after induction treatment. Of these, 21 patients (29%) received a subsequent line of systemic therapy, while 16 patients (22%) continued treatment beyond progression due to clinical benefit. LAT was administered in 10 patients continuing nivolumab and ipilimumab beyond progression. The addition of LAT to immunotherapy improved the median duration of post-progression treatment from 5.6 months to 8.7 months. Results from cohort 1 are pending [97].

The NRG-LU002 trial (NCT03137771) is a phase II/III study that aimed to evaluate the impact of adding LAT to maintenance systemic therapy in patients with up to three metastatic sites and no progression after initial systemic therapy. Participants were randomized 2:1 to receive either maintenance systemic therapy combined with LAT (SBRT or surgical resection) or systemic therapy alone. The primary endpoint was PFS rate at 6 and 12 months, and approximately 90% of patients enrolled received immunotherapy as part of induction therapy. PFS in the LAT arm was comparable to that in the standard therapy arm, with a 12-month PFS rate of 51.0% vs. 48.0%, respectively (HR 0.93, 95% CI 0.65–1.31). With a median follow-up of 21.9 months, the 24-month OS rate was 58.1% in the combination arm vs. 54.1% in the control arm (HR 1.05, 95%CI 0.70–1.56). Limitations of the trial included the variability in systemic therapy regimens, absence of biomarker enrichment, and the inclusion of patients with polymetastatic disease that presented induced oligometastatic disease. This study has closed recruitment after this planned interim analysis [98].

The ETOP 14–18 CHESS trial (NCT03965468) is a prospective, single-arm, international phase II study that aims to assess the safety and efficacy of a multimodal treatment approach in synchronous OMD-NSCLC. All patients were staged with ^18^F-FDG PET-CT and brain MRI. The induction phase consisted of four to six cycles of chemoimmunotherapy (durvalumab, carboplatin, and paclitaxel) with upfront LAT (SBRT or surgical resection) to all metastatic sites. Patients without progression at the 3-month restaging by ^18^F-FDG PET-CT underwent LAT to locoregional disease, followed by continuation of durvalumab for up to 1 year. The primary endpoint was PFS at 12 months, with an improvement from ≤25% to ≥50% under trial treatment. Among the first 42 evaluable patients, the 12-month PFS rate was 33% (primary endpoint not met). Median PFS was 9.4 months, and progressive disease occurred in 22% of patients after induction therapy. Distant progression was the most common failure mode, occurring in 65% of patients. Median OS was 18.2 months, while the OS rate at one year was 74.9%. Despite not meeting its primary endpoint, the promising survival rates have led to exploring treatment intensification with the addition of tremelimumab during the induction phase [99].

Results from the CHESS and NRG-LU002 trials challenge prior assumptions about the benefits of LAT in OMD-NSCLC, underscoring the systemic nature of the oligometastatic state. Having this in consideration, we currently wait for the results of other ongoing phase 3 clinical trials before we may adopt LAT as a standard of care: NIRVANA LUNG (NCT03774732), TARGET-02 (NCT05278052), ANDROMEDA (NCT06141070), CORE (NCT02759783), STEREO-OS (NCT03143322), among others.

*OMD-NSCLC with targetable molecular alterations:* Patients with targetable molecular alterations, such as EGFR or ALK, were either excluded or underrepresented in historical OMD-NSCLC clinical trials [87,88]. Implementing LAT in OMD-NSCLC patients with TMA may eradicate resistant cell clones and prolong the efficacy of TKIs [100,101,102].

The SINDAS trial (NCT02893332), a randomized, phase III study, aimed to address the benefit of LAT associated with targeted therapy in patients with *EGFR*-mutated synchronous OMD-NSCLC, with a maximum of five lesions. Absence of brain metastases was mandatory. Patients were randomized 1:1 to receive first-generation EGFR inhibitors (gefitinib, erlotinib, or icotinib) with or without SBRT prior to starting TKI. A total of 133 patients were included. Median PFS and OS were improved in the TKI + SBRT arm: median PFS of 20.2 vs. 12.5 months, HR 0.22 (95CI% 0.17–0.46); median OS 25.5 vs. 17.6 months, HR 0.44 (95%CI 0.28–0.68) [103].

The ATOM trial (NCT01941654) was a single-arm, phase II study that included patients with oligo-induced OMD-NSCLC harboring common EGFR mutations. Patients were eligible if ≤4 PET-avid lesions were identified after 3 months of first- or second-generation TKI therapy. LAT included SBRT or surgery. The primary endpoint was 1-year PFS, reaching a rate of 68.8%, median PFS of 15.2 months, and median OS of 44.3 months. In this case, central nervous system metastases were allowed if previously treated and stable [104].

The NRGO-002 trial (ChiCTR-IIR-16007769) was a multicenter, randomized, controlled, phase III study assessing the benefit of thoracic radiotherapy (TRT) on survival in patients with EGFR-mutated OMD-NSCLC treated with icotinib as first-line treatment. Patients randomized to TRT received a total dose of 60 Gy in 30 daily fractions to the primary lung tumor and positive regional lymph nodes. A total of 118 patients were randomized in a 1:1 fashion to icotinib or icotinib + TRT. Median PFS improved in the TRT + icotinib arm: 17.1 vs. 10.6 months, HR 0.57 (95%CI 0.34–0.843; *p* = 0.04). Median OS was also improved: 34.4 vs. 26.2 months, HR 0.62 (95%CI 0.41–0.96; *p* = 0.029). This benefit was accomplished at the expense of a higher incidence of severe (grade 3/4) adverse events, with around 7% of patients experiencing severe radiation esophagitis and 5% severe radiation pneumonitis. The proportion of patients with five or fewer metastases was higher in the TRT plus icotinib arm, as well as patients with up to one organ affected by metastases. Therefore, it is discussed whether the separation of the PFS curves could be in relation to an imbalance in prognostic factors between groups [105].

The most important limitations of these trials were not implementing a third-generation TKI (i.e., osimertinib) as a systemic therapy backbone. In that sense, the LUNG-SORT trial (NCT04764214) evaluated the efficacy and safety of consolidative SBRT for oligo-residual disease (ORD) in patients receiving a third-generation TKI. Extracranial ORD was defined as residual tumors limited to three organs and five lesions after effective third-generation EGFR-TKIs in patients without baseline brain metastases and those with complete intracranial response. Meanwhile, cranial ORD was defined as brain metastases limited to three lesions with a largest diameter of ≤3 cm after effective third-generation EGFR-TKIs among those with residual brain metastases and without extracranial progressive disease. The primary endpoint was PFS. A total of 61 patients were included, all being able to receive SBRT. Median PFS was 29.9 months, with a 1-year and 2-year PFS rate of 91.6% and 53.4%, respectively. Only four patients suffered from grade ≥ 3 treatment-related adverse events (one case each of pneumonitis, esophagitis, leukopenia, and cranial irradiation necrosis). A propensity score matched comparison was conducted with a contemporary cohort of patients, revealing improvement in median PFS in patients receiving TKI + SBRT: HR 0.46 (80%CI 0.20–0.61); *p* = 0.002) [106].

Contrary to *EGFR*, trials of OMD-NSCLC in patients with *ALK* fusions or other TMAs (i.e., *BRAF*, *ROS1*, *NTRK*, *RET*, etc.) are lacking. BRIGHTSTAR (NCT03707938) is a single-arm phase 1 trial that included patients with oligo- or polymetastatic (>3 sites) NSCLC with ALK alterations. Patients received brigatinib for 8 weeks, followed by RT and/or surgery if at least one residual site of disease was available for LAT. A total of 34 patients have been included; 82% had polymetastatic disease at study entry. The objective response rate after induction therapy was 79%, and 32 completed LAT (5 patients underwent surgery). The 24-month PFS rate was 80%, and lower disease burden at disease presentation and after induction was associated with improved PFS. This trial shows that LAT is also feasible in patients with TMA other than EGFR [107]. Ongoing trials for OMD-NSCLC and TMA include OPTALK (NCT06620835), NORTHSTAR (NCT03410043), OMEGA (NCT03827577), TARGET-01 (NCT05277844), and HALT (ISRCTN53398136).

A summary of clinical trials is shown in Table 2.

Taking into consideration the hypes in the history of OMD-NSCLC’s management, the ESTRO/ASCO clinical practice guideline on treatment of OMD-NSCLC recommends starting with systemic therapy for at least 3 months, after which treatment response should be assessed, and only in patients without disease progression should definitive LAT be offered [55].

## 8. Conclusions

An impressive surge has occurred in the development of therapeutic strategies for patients with OMD-NSCLC. That said, there are conflicting results when we have tried to implement these strategies, especially in patients receiving immunotherapy as part of the systemic therapy backbone. Therefore, discussion of each case in a multidisciplinary committee becomes imperative [108]. Future steps include carrying out more translational studies to further learn about the biology of the oligometastatic state, implementing standard definitions when designing clinical trials to homogenize results, and exploring different biomarkers that may aid us in selecting appropriate candidates for radical therapies.

## Figures and Tables

**Table 1:** Summary of different OMD-NSCLC definitions.

Concept	Reference	Definition
Synchronous or de novo OMD	Fleckenstein et al. 2016 [14]	OMD at the time of the initial diagnosis.
	Nguyen et al. 2022 [15]	OMD at the time of diagnosis in which a primary tumor and limited number of metastases are detected simultaneously.
Sync-oligometastases	Niibe et al. 2012 [16]	Patients with cancer have ≤5 metastatic or recurrent lesions with active primary lesions.
Oligo-recurrence	Niibe et al. 2010 [17]	Cancer patients with 1–5 metastatic or recurrent lesions that could be treated by local therapy with controlled primary lesions.
	Guckemberger et al. 2020 [11]	Growth of limited numbers of metastatic deposits in patients off systemic therapy.
Metachronous OMD	Fleckenstein et al. 2016 [14], Nguyen et al. 2022 [15]	Oligometastatic recurrence during the course of disease at least three months after the initial diagnosis, as a state of metachronous limited recurrence.
Oligo-progressive disease	Pembroke et al. 2018 [18]	Few lesions progress on a background of widespread but stable metastatic disease.
	Nguyen et al. 2022 [15]	Few lesions (≤5) progressing in the background of otherwise stable OMD or stable polymetastatic disease.
	Campo et al. 2016 [19]	Patients with disseminated disease at diagnosis respond to systemic treatment, remaining stable while one or a limited number of metastases progress during systemic therapy.
	Guckemberger et al. 2020 [11]	Patients with oligometastases receiving active systemic treatment with progression of disease in a limited number of existing and/or new sites.
Oligo-persistent disease	Laurie et al. 2019 [20]	Oligometastatic state that, after systemic therapy, either persists or is induced from a more widely metastatic state.
	Guckemberger et al. 2020 [11]	Stable disease or partial response of the existing limited disease to therapy.

**Table 2:** Summary of phase II-III clinical trials in patients with OMD-NSCLC with or without TMA.

Study (Ref.)	Phase/Design	Population	Systemic Therapy	LAT Modality	Main Outcomes	Key Notes
OMD-NSCLC trials without targeted therapy						
De Ruysscher et al. (2012) [85]	Phase II, single-arm	Synchronous OMD (≤5 lesions), *n* = 39	CCRT (54%), sequential CT (38%)	Surgery, RT, SRS to mets	mPFS: 12.1 mo; mOS: 13.5 mo	No immunotherapy upon progression
Gomez et al. (2016/2019) [86,87]	Phase II, randomized	Post-induction (CT or TKI), *n* = 49	4 cycles CT or ≥3 mo TKI	SABR, surgery ± RT	mPFS: 11.9 vs. 3.9 mo; mOS: 41.2 vs. 17.0 mo	Trial stopped early; mostly EGFR/ALK WT
Iyengar et al. (2018) [88]	Phase II, randomized	Post-CT (4–6 cycles), EGFR/ALK WT, *n* = 29	Maintenance CT	SABR to all lesions	mPFS: 9.7 vs. 3.5 mo; OS NR vs. 17 mo	Trial stopped early; similar toxicity
LONESTAR (NCT03391869)	Phase III, ongoing	IO-naïve or post-induction PD	Nivolumab + ipilimumab	SABR, surgery	mPost-PD treatment: 8.7 vs. 5.6 mo	Preliminary results (ASCO 2022)
NRG-LU002 (NCT03137771)	Phase II/III, randomized	≤3 mets post-induction, *n* = 196	Chemo ± IO (90% received IO)	Surgery or SBRT	12-mo PFS: 51% vs. 48%; 2-year OS: 58.1% vs. 54.1%	Negative trial; no biomarker stratification
CHESS (ETOP 14–18)	Phase II, single-arm	Synchronous OMD, *n* = 42	Induction chemo-IO (durvalumab), then maintenance	LAT to mets + thoracic RT	12-mo PFS: 33%; mPFS: 9.4 mo; mOS: 18.2 mo	Primary endpoint not met; encouraging signals
OMD-NSCLC trials with targeted therapy						
SINDAS (NCT02893332)	Phase III, randomized	EGFR-mutated synchronous OMD (≤5), *n* = 133	First-gen EGFR TKI	SBRT	mPFS: 20.2 vs. 12.5 mo; mOS: 25.5 vs. 17.6 mo	Brain metastases excluded
ATOM (NCT01941654)	Phase II, single-arm	EGFR-mutant oligo-induced OMD	TKI x3 mo → LAT	SBRT or surgery	1-year PFS: 68.8%; mPFS: 15.2 mo; mOS: 44.3 mo	Brain mets allowed if stable/treated
NRGO-002	Phase II, randomized	EGFR-mutant OMD	Icotinib ± thoracic RT	Thoracic RT (60 Gy)	mPFS: 17.1 vs. 10.6 mo; mOS: 34.4 vs. 26.2 mo	More toxicity in RT arm (pneumonitis and esophagitis)
LUNG-SORT (NCT04764214)	Phase II, single-arm	EGFR-mutant ORD post-osimertinib	Osimertinib	SBRT to residual disease	mPFS: 29.9 mo; 1-year PFS: 91.6%	Propensity score-matched analysis showed benefit
BRIGHTSTAR (NCT03707938)	Phase I	ALK+ NSCLC, oligo/polymetastatic	Brigatinib induction x8 wks	LAT to residual disease	24-mo PFS: 80%	82% polymetastatic at baseline; LAT guided by response

Abbreviations: OMD: oligometastatic disease. LAT: local ablative therapy. CT: chemotherapy. CCRT: concurrent chemoradiotherapy. IO: immunotherapy. TKI: tyrosine kinase inhibitor. SABR: stereotactic ablative radiotherapy. RT: radiotherapy. SRS: stereotactic radiosurgery. mPFS/mOS: median progression-free survival/overall survival. NR: not reached.
